# On the coloniality of global public heath

**DOI:** 10.17157/mat.6.4.761

**Published:** 2019-12-16

**Authors:** Eugene T. Richardson

**Affiliations:** Harvard Medical School

**Keywords:** Ebola, coloniality, mistrust, epistemic violence, hermeneutic injustice, symbolic reparations

## Abstract

The continued inordinate demise from communicable pathogens in the global South is not the result of an intractable problem thwarting our best efforts to prevent and cure disease; we have the means. Rather, as an accomplice to contemporary imperialism, public health manages (as a profession) and maintains (as an academic discipline) global health inequity. It does this through ‘bourgeois empiricist’ models of disease causation, which serve protected affluence by uncritically reifying inequitable social relations in the modern/colonial matrix of power and making them appear commonsensical.

## Introduction: The epidemiologist as fabricant

In the novel *Cloud Atlas*, the reader is presented with a future in which clones known as ‘fabricants’ work as cheap labor in a hypercorporate dystopia (Mitchell 2004). Their minds are stunted via chemical manipulation to prevent them from rebelling or performing radical acts. After twelve years of service, they are promised retirement in Hawaii, but in actuality are butchered and recycled as food for other fabricants.

Somewhat analogously, those who seek training in objectivist epidemiology are programmed for a life of unradical (in other words, corporate-friendly) approaches to parsing social phenomena. The major difference with the fabricant is that they will be fed secondary data sets, which can be thought of as digested bits of the subaltern (insofar as research subjects in the global South are reaped as academic fodder without any benefit accruing to them).

As I argue below, the continuation of inordinate mortality from communicable pathogens in the global South is not the result of an intractable problem thwarting the global community’s best efforts to prevent and cure disease; we do have the means. Rather, moral detachment in academic fabricants who are subservient to protected affluence – a subservience sculpted by the categories and methods supplied in academic training – allows for anemic approaches to the study and achievement of global health equity (Levins and Lewontin 1985; Pogge 2008).

## Ebola and the narrative of mistrust

They who have put out the people’s eyes, reproach them of their blindness.

– John Milton, *Apology for Smectymnuus*

Early in 2019, researchers from the Harvard School of Public Health published findings from a population-based survey in the Democratic Republic of the Congo (DRC), in which they concluded that people refused to seek formal medical care or accept vaccines during the 2018 (still ongoing) Ebola outbreak because they did not believe Ebola virus was real (Vinck et al. 2019). The findings were picked up quickly by international news outlets^1^ and helped reinforce a narrative that sufferers of Ebola virus disease (EVD) have their false beliefs in conspiracy theories to blame for the spread of the outbreak. In the following months, I watched how this narrative of mistrust sedimented as a cultural claim of causality among the media, scholars, DRC ministry officials, other responders in North Kivu/Ituri provinces, and the World Health Organization (WHO).

### Coloniality

Gather ye facts as ye may.

– Herrick/Richardson, *To the Epidemiologists, too Make Much of Justice*

My interest here is not to refute the authors’ reported results – they did gather facts^2^ – but rather to expose the epistemic violence such fact gathering commits (through the analytic omission of the power relations that determine levels of trust in the postcolony [Mbembe 2001]). I submit that these types of ‘scientific’ analysis draw from a mental map whose contours are shaped by coloniality, which can be defined as the matrix of power relations that persistently manifests transnationally and intersubjectively despite a former colony’s achievement of nationhood. As a conceptual apparatus, ‘coloniality’ attempts to capture the racial, political-economic, social, epistemological, linguistic, and gendered hierarchical orders imposed by European colonialism that have transcended ‘decolonization’ and continue to oppress in accordance with the needs of pan-capital (economic and cultural/symbolic) accumulation (Quijano 2000). Examples include institutionalized racism (Dubois 1987), religious discrimination, economic exploitation (Nkrumah 1965; Rodney 1972; Amin 1973), control of gender and sexuality (Fraser 1989; Stoler 1995; Butler 2006; Lugones 2007), and dominion over subjectivity and knowledge (epistemology and education) (Mignolo 2007). As Grosfoguel (2007, 219) puts it, ‘the heterogeneous and multiple global structures put in place over a period of 450 years did not evaporate with the juridical-political decolonization of the periphery over the past 50 years. We continue to live under the same “colonial power matrix”. With juridical-political decolonization we moved from a period of “global colonialism” to the current period of “global coloniality”’.

As wa Thiong’o (1986, 16) has taught, ‘Colonialism imposed its control of the social production of wealth through military conquest and subsequent political dictatorship. But its most important area of domination was the mental universe of the colonized, the control, through culture, of how people perceived themselves and their relationship to the world’. This was accomplished through the construction of narratives that produced Africans as racial subjects and sites of savage exteriority, setting them up for moral disqualification and practical instrumentalization (Mbembe 2017). These narratives, which are essentially story technologies invested with (social-)scientific legitimacy (Said 1979; Foucault 1979; Gutiérrez 1974), continue as the logic of contemporary coloniality.

This think piece aims to demonstrate how modern social scientists – like fabricants – have had their moral outlooks stunted by such logic, which then delimits how they gather facts. After discussing counterhegemonic ways of interpreting health phenomena, I conclude with ways to delink knowledge production from the colonial matrix of power.

### Bourgeois empiricism and hermeneutic injustice

At best, studies like the one conducted by Vinck and colleagues (2019) are analytically irresponsible in their collapsing of trust and belief with health-seeking practices, as well as their insensitivity to the *longue durée*. At worst, these ahistorical analyses are a form of neoliberal propaganda that serves to efface the determinants of mistrust that Congolese conspiracy theories are indeed critiquing. Were we to appreciate mistrust as an inclination, a cognitive tendency (Mills 2007), or a structured disposition (in other words, habitus) towards eluding depredation – not simply as a rational calculation based on ‘misinformation’ – then its capacity as a mediator in a determinative web of human rights abuses that stretch back in time and link the DRC to distant continents^3^ could rise to the level of common sense.

Instead, however, such studies trace the causal pathway of Ebola transmission in this way: ‘lack of trust -> non-compliant actors -> Ebola outbreak propagation’, thereby omitting its historical and geopolitical antecedents. In so doing, epidemiologists actively reinscribe – and therefore participate in (Young 2011) – centuries-old racial hierarchies that have underscored and legitimated the (neo)colonial project, under the guise of objective ‘empiricism’ (Richardson and Polyakova 2012).^4^ In other words, through discursive hegemony (Gramsci 1971; Schwartz et al. 2016), they prevent structural determination from becoming commonsensical by dominating how people – including voting citizens and policy makers in the global North – perceive and interpret health phenomena.

Such interpretations therefore commit ‘hermeneutic injustice’ (Pohlhaus 2012), meaning malfeasance in the way one interprets what one sees, by 1) denying conspiracy theories as valid critiques of the coloniality of power and 2) recycling cultural claims of causality that mystify more than one hundred years of colonial atrocities and predatory accumulation as explications (Farmer 2001; Richardson, Barrie, et al. 2016).

From my previous experience taking care of patients and interviewing survivors and vaccine candidates during the Ebola outbreaks in Sierra Leone, Liberia, and the DRC, I have come to appreciate such conspiracy theories as a practical logic of engagement with the *Maafa*, which is the Swahili word for ‘disaster’ or ‘great tragedy’. ‘*Maafa*’ is used to evince the history and ongoing effects of the African holocaust of slavery, colonialism, and neocolonialism (Ani 1994; Wittmann 2016). Accordingly, these conspiracy theories coalesce with other postcolonial critiques to become truth claims that demand reparations and redistributive justice, not bourgeois empiricism – which can be defined as a gathering facts ‘that hide[s] behind scientific objectivity to perpetuate dependency, exploitation, racism, elitism, [and] colonialism’ (Levins and Lewontin 1985, 4) – or the crisis caravan, that flotilla of NGOs and development organizations that move from emergency to emergency, ‘scattering aid like confetti’ (Polman 2010, 157).

## Immodest Models

‘Aid’, therefore, to a neocolonial State is merely a revolving credit, paid by the neocolonial master, passing through the neocolonial State and returning to the neo-colonial master in the form of increased profits.

– Kwame Nkrumah, *Neocolonialism, The Last Stage of Imperialism*

The unjust gathering of facts is not limited to empirical observation but includes the choice of variables used in epidemiological modeling as well. For example, Bendavid and Bhattacharya (2014) used difference models to demonstrate that development assistance for health (DAH, a type of aid) was associated with improvements in health indicators in the countries receiving it. Their results were published in the prestigious *JAMA Internal Medicine* and helped buttress the core tenet of neocolonialism summarized by Nkrumah above.

I built a somewhat similar computational model:

$$H_{t}=\beta_{0}+\beta_{1}DAH_{t}+\beta_{2}GDPpc_{t}+\beta_{3}Urban_{t}+\beta_{4}TFR_{t}+\beta_{5}IFF_{t}+\varepsilon_{t}$$

where H is the recent under-five mortality in a country, DAH is the logarithm (log) of the total health aid that was received from 1970–2008, GDPpc is their recent gross domestic product per capita, Urban is their percent urbanization, TFR is their recent total fertility rate, and ε is an error term. I then added a variable (not included in the *JAMA* analysis) for *Illicit Financial Flows* (IFFs), which can be defined as illegal movements of money or resources from one country to another that reduce the amount of capital and revenue available within a country to develop public services such as health care systems (Jubilee Debt Campaign 2017). Subsequent linear regression models demonstrated that decreases in under-five mortality associated with DAH were nearly offset by increases in under-five mortality associated with IFFs. I further found that the log of total health aid was highly correlated with the log of IFFs (*r* = 0.65), raising the question of whether DAH is used to disguise illicit financial flows.^5^

### Symbolic violence

‘Superspreaders’ Caused More Than 60% of Infections during the Ebola Epidemic.

– Peter Dockrill, *ScienceAlert*

If superspreading had been completely controlled, almost two-thirds of the infections might have been prevented, scientists said.

– Lena Sun, *Washington Post*

These quotes represent the media ramifications of another modeling study published by Princeton investigators in the *Proceedings of the National Academy of Sciences* (PNAS). Their computer simulations ‘found’ that ‘superspreaders’ played a key role in sustaining onward transmission of the Ebola epidemic in West Africa, and that these individuals were *responsible* for a significant proportion of infections (Lau et al. 2017). (In contemporary EpidemiologySpeak, ‘superspreaders’ are defined as infected individuals who disproportionately transmit pathogens to susceptible people.) Elsewhere, my colleagues and I argue that another descriptor, ‘PPE^6^-bereft-care-nexus’, is a more just term, since it highlights the fact that EVD is a caregivers’ disease (Farmer 2015) that thrives in underdeveloped and historically plundered regions, and that the use of terms such as ‘superspreader’ unjustly implicates marginalized individuals as culpable for the spread of disease (Richardson, Barrie, et al. 2017).

**Figure 1.**
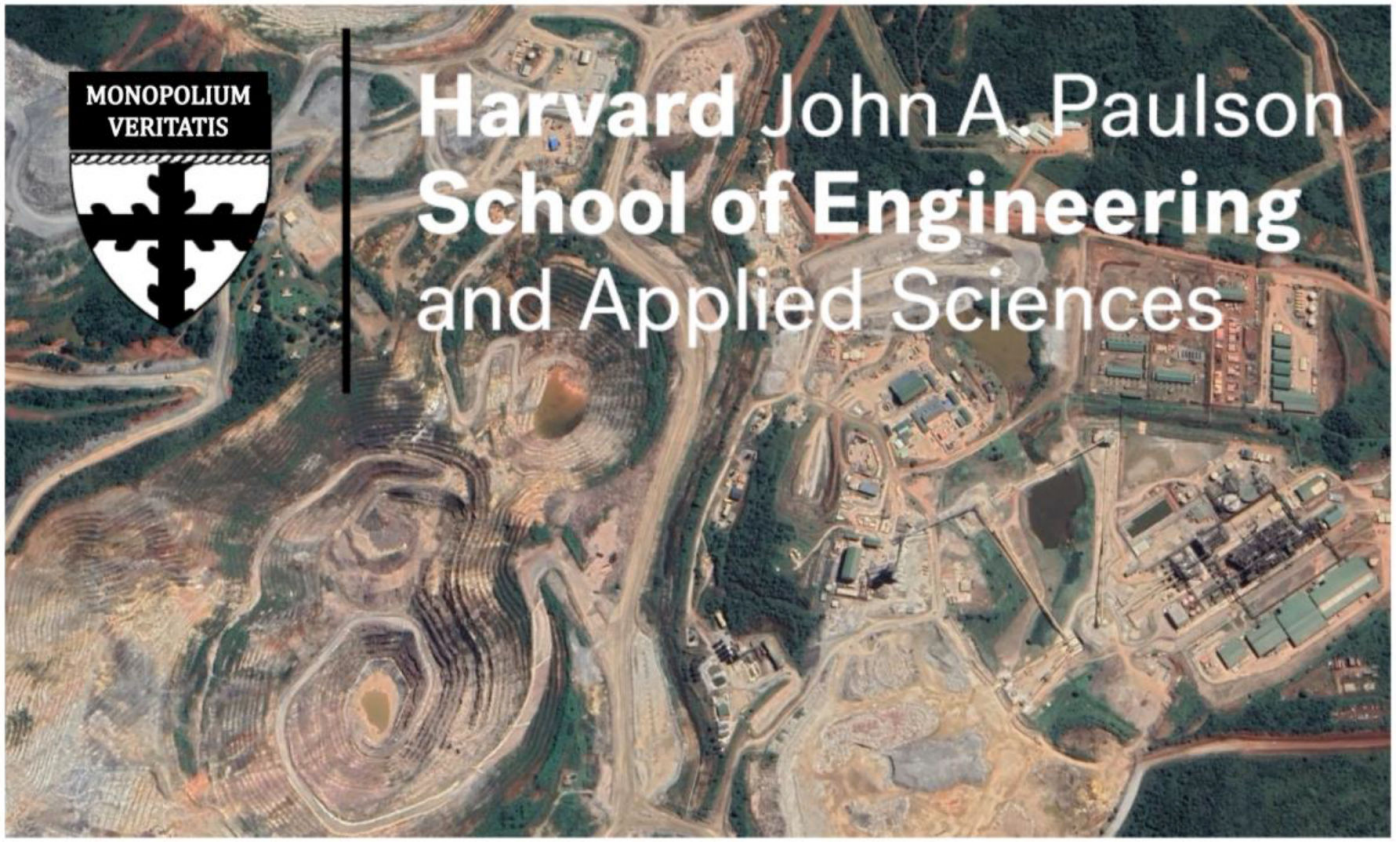
Superspreading nexus. Satellite image of the Kibali gold mine in DRC superimposed with a revised logo of the John A. Paulson School of Engineering and Applied Sciences (SEAS) at Harvard University (translation from Latin: Monopoly on truth) (image source: Google Earth). Kibali is co-owned by AngloGold Ashanti (45 percent), Barrick Gold Corporation (45 percent) following its merger with Randgold Resources Limited, and Société Minière de KiloMoto (SOKIMO) (10 percent), a state-owned gold mining company. In 2015, John A. Paulson, the largest single shareholder of AngloGold Ashanti, donated US$400 million – the largest gift in the university’s history – to support SEAS (J. Sachs 2016).

It can be useful to think of *PNAS, JAMA, The Lancet* and other high-impact journals as validating the types of discourse that the ‘scientific community’ accepts and makes function as true (Foucault 1979), or, as instruments for enforcing meaning (Haraway 1991). If an accepted discourse causes harm or sustains relations of domination, it can be deemed as causing symbolic violence. By this definition, the Princeton investigators commit symbolic violence by authenticating the use of ‘superspreader’ as a synchronic epidemiological descriptor of bounded individuals as agents of disease transmission (Kelly et al. 2018), without acknowledging how sociohistorical forces become embodied as EVD (Richardson, Morrow, et al. 2016). In this way, the authors unintentionally (since they are no doubt compassionate global health advocates) function as ‘transfer mechanisms’ (Keshavjee 2014) for the neoliberal ideology of predatory accumulation (Kieh 2017), in essence diverting the public’s gaze from legacies of the *Maafa*, colonialism, indirect rule, structural adjustment, illicit financial flows, and extractive foreign companies (Césaire 1972; Kim et al. 2002; Jubilee Debt Campaign 2017; Hickel 2018; Frankfurter et al. 2018; Burgis 2016; Richardson and Fallah 2019). *Pace* Lau and colleagues, one can read ‘residual imperialist propensities’ (Said 1993) in their work, for example in statements such as, ‘Understanding superspreading can facilitate devising individually targeted control measures, which may outperform population-level measures’ (Lau et al. 2017, 2337). This could be roughly interpreted as ‘earmark US$3 billion to stop human superspreaders, not corporate ones’ (see figure 1).

### Ebola vaccines and the ideal speech situation

In his early philosophical work, Habermas (1978) described the ideal speech situation as a rational exchange of dialogue where unconstrained consensus on truth claims can be achieved, that is, where factualness is not distorted by domination, ideology, and repression. If we examine the above narratives of superspreaders and mistrust, we find that ideological distortions preclude ideal speech. Instead, it may be more revealing to view these narratives, in Foucauldian fashion, as contested sites of power that help us ‘make sense of the insidious, often almost invisible nature of ideology today’ (Agger 2006, 96). By comparing epidemiologists’ accounts of disease ‘causation’ with those of EVD patients, survivors, and their close contacts ‘we allow [*sic*] the anthropologist’s informants the privilege of explicating and publicizing their own criticisms of the forces that are affecting their society – forces which emanate from ours’ (Taussig [1980] 2010, 6).

### Subaltern empiricism: Why are we sick?

The interviews I conducted with EVD patients, survivors, and their close contacts provide different ways of understanding the vocabulary employed by epidemiologists to describe Ebola virus transmission. For example, after discussing the concept of ‘superspreader’ with a number of people affected by the outbreak, not one agreed using the term to describe individuals was appropriate. Some felt their national governments should be deemed superspreaders because of endemic corruption; others felt foreign corporations were to blame. In Liberia, one man remarked, ‘Firestone was the superspreader, since their efforts prevented us from getting a tire factory’; others discussed the legacy of the trans-Atlantic slave trade or *Maafa*.

In addition, during an Ebola containment campaign in Ituri Province where my WHO colleagues and I were able to vaccinate only eight people in village of more than two hundred, I asked rural Congolese directly about the mistrust narrative. The replies were similar to that recorded by the journalist Maxmen (2019): ‘People think this is just another thing brought from outside to kill [us]’.

A separate coterie of individuals who refused the vaccine agreed that AngloGold Ashanti, one of the largest gold-mining companies in the region, had helped set the conditions for the outbreak:^7^ ‘We have nothing to show for all this wealth underground’, a deputy town chief offered. Another chimed in, ‘They pay rebels’. Such claims have been substantiated: in ‘Blood and Treasure: Why One of the World’s Richest Countries Is Also One of Its Poorest’, Hochschild (2010) describes how AngloGold Ashanti made ‘payments to the warlord who controlled Mongbwalu [in Ituri province]…also providing him and his entourage rides in company planes and vehicles, and a house on its concession’.

After the person voiced the charge that AngloGold Ashanti paid rebels, I asked the group, ‘What if I told you that my university accepted hundreds of millions of dollars from one of the owners of that company?’^8^ ‘Then they’re part of it, too’, a part-time miner replied.

In short, there are ways of gathering facts that serve the interests of the subaltern, and such ‘border gnosis’^9^ is integral to dismantling coloniality. But what a person says and what a person does are often very different things. My World Health Organization colleagues and I rarely received responses to the question, ‘Why aren’t you interested in the vaccine?’ (which of course was asked in translation). People often demurred, and when pressed would sometimes offer the conspiracy theories described by Vinck and colleagues (2019). (This reminded me of Evans-Pritchard [(1937) 1976, 24]: ‘For if Azande cannot enunciate a theory of causation in terms acceptable to us they describe happenings in an idiom that is explanatory’.) I might add to this early anthropological insight that ‘idiom’ include an interlocutor’s habitus. In other words, border gnosis consists of both discursive knowledge and actual practice.

### Habitus

In the case of Ebola vaccine acceptance, Kasereka and colleagues (2019, 2174) report that there was high community acceptability for the vaccine in DRC (‘72% of unvaccinated community controls would wish to be vaccinated if supply were available’). This did not play out in actual practice, however. When supply was available to Ebola patients’ close contacts, the large majority of them declined to be vaccinated.

One could view this response as part of a structured disposition for eluding depredation, described earlier. In other words, vaccine refusal is a form of border gnosis that represents the reactivation of a sedimented dialogic (Bakhtin 1981) of historical rapine and resistance.

### Decoloniality

The distinctions between epidemiology as an unbiased scholarly endeavor and epidemiology as an accomplice to contemporary imperialism are a matter of how one gathers facts (Ake 1982; Cerón 2019). This quintessential discipline of public health is involved in worldly, historical circumstances that it has tried to conceal behind a speciously rigorous scientism (Said 1979; Adams 2016). Similar to Giridharadas’s (2018) analysis of social entrepreneurship in *Winners Take All*, the modest improvements in well-being offered by the right hand of global public health science disguise what global elites and their looting machines (Burgis 2016) take with the left (Hickel 2018). One could also posit a similarity between Chipato’s (2019) description of the aid industry’s co-optation of Zimbabwean activists in the 1990s (‘the NGOization of political protest’) and the relegation of political radicals to schools of public health after the failure of the American Left to act as a transformative political-ideological social force (Pollack 2015).

As fabricants, those relegated to schools of public health have been programmed with bourgeois empiricist (in other words, deradicalized) approaches to health phenomena. And as a consequence, they are prone to committing symbolic violence on one front and colluding in economic injustice on the other. By seeing these dual forms of violence as the means by which the modern/colonial racist/patriarchal system (Grosfoguel 2011) continues to operate, we can justify calls for an Epistemic Reformation.

### The Epistemic Reformation

Compared to the Catholicism that stunted the minds of Europeans throughout the Dark Ages, until Luther posted his *Ninety-five Theses* in 1517, the coloniality that has permeated our thinking ever since is more encompassing (and global) in its reach. As such, a similar democratization of knowledge – an Epistemic Reformation – must occur, specifically by devolving scientific authority from ‘centres of calculation’ (Latour 1988) in the global North.

The practice of counter-hegemonic epidemiology as a form of epistemic reformation is not novel (Radical Statistics Group n.d.; Berkman, Kawachi, and Glymour 2014; Avilés 2001; Breilh 2003); academics and collective health specialists from the global South have been grappling with it for decades (Breilh 2008).^10^ Lowes and Montero (2017) provide a degree of epistemic reconstitution with their study of medical interventions aimed at eradicating human African trypanosomiasis (sleeping sickness) in French Equatorial Africa. Utilizing thirty years of archival data from French military archives of five former colonies, including Congo-Brazzaville, they demonstrate that increased exposure to colonial medical campaigns – characterized by forced lumbar punctures and treatment with aminophenyl arsonic acid (atoxyl), a somewhat effective arsenic compound that left 20 percent of patients blind – is correlated with lower levels of trust in medicine today (Lowes and Montero 2018). While they recapitulate the conflation of trust and health-seeking behavior exemplified in the work by Vinck and colleagues, their study is an example of how public health research can begin to ‘parametrize’ (turn into variables for computational modeling purposes) historical and structural forces in shaping populations’ dispositions towards medicine and health care.

It seems logical that a similar dynamic of mistrust was engendered across the Congo River in the Belgian colony, where, during the first half of the twentieth century, people who were suspected of having sleeping sickness were detained in camps (staffed by Catholic nuns) that were notable for toxic therapy, poor conditions, lack of food, and the permanent separation of patients from their families, all under armed guard (Headrick 2014). Like Alsan and Wanamakers’ (2017) excellent study of contemporary mistrust related to the egregiously unethical Tuskegee experiments, these legacies of colonial medicine remind us that mistrust is not formed in a vacuum, that is, ‘cultural’ beliefs do not overdetermine health-seeking practices.

## Conclusion

While recognizing the devastating impact of material deprivation on the health of populations, this paper responds to Boaventura de Sousa Santos’s (2014) claim that global social injustice is by and large epistemological injustice and that there can be no global social justice without addressing symbolic violence. By tracing human rights failings to the impoverished discursive infrastructure of objectivist epidemiology (Good 1994; Pogge 2008; Kleinman 1994), we could transform global health by transforming its representations (Richardson, Kelly, et al. 2017; Bourdieu 1981; Cornwall and Eade 2010; W. Sachs 2009; Freire 2000).

## Footnotes

1 The Associated Press, for example, reported: ‘Researchers said their study showed more precisely how individuals’ misinformed views about Ebola were undermining the response and helping to spread the deadly virus’ (Larson 2019).

2 Nor am I interested in the social construction of facts (Berger and Luckmann 1967); as a pragmatist (or populist), I accept there are different ways of parsing phenomena that all vie equally for epistemic status.

3 These include the large-scale atrocities committed by King Leopold and Belgium in the late nineteenth and early twentieth centuries (Hochschild 1998), Cold War clientelism and resource theft (Nzongola-Ntalaja 2013), and regional genocidal wars whose roots lie deep in the colonial history of ethnic identity construction and mobilization (Stearns 2011), to name but a few.

4 ‘The question is no longer the positivist question of whether representation is accurate, copying a reality that is extrasymbolic … but what reality is being constructed, by whom, for whom, for what political purpose, and to what political effect’ (Biersack and Greenberg 2006).

5 See Eugene Richardson, *Epidemic Illusions* (MIT Press, forthcoming) for an expanded exploration of the model.

6 ‘PPE’ stands for ‘personal protective equipment’, worn by professional Ebola caretakers to protect themselves from infection.

7 My colleagues and I have analyzed a similar dynamic in Sierra Leone (Frankfurter et al. 2018).

8 ‘AngloGold Ashanti mined more than $1.5 billion worth of gold in neighboring Tanzania between 2000 and 2007, but only 9 percent of that money has remained in the country as taxes or royalties. Where do the profits go instead? A good chunk comes to the United States, for even though the company is based in South Africa, its largest single shareholder – hedge fund billionaire John Paulson – lives on the Upper East Side and summers in the Hamptons. He owns 12 percent of the company, and a number of other Americans have shares’ (Hochschild 2010).

9 As Mignolo (2012, 11) teaches, ‘Border gnosis as knowledge from a subaltern perspective is knowledge conceived from the exterior borders of the modern/colonial world system. … [It] is conceived at the conflictive intersection of the knowledge produced from the perspective of modern colonialisms (rhetoric, philosophy, science, [epidemiology]) and knowledge produced from the perspective of colonial modernities in Asia, Africa, and the Americas/Caribbean’.

10 As part of this effort, we have formed a Lancet Commission on Reparations and Redistributive Justice that has a section dedicated to symbolic reparations; see https://projects.iq.harvard.edu/lancet-reparations/home.
